# MELAS Syndrome Presenting with Hypertrophic Cardiomyopathy and Advanced Heart Failure: A Multisystem Diagnostic Challenge

**DOI:** 10.3390/jcm15031109

**Published:** 2026-01-30

**Authors:** Jozef Dodulík, Marie Lazárová, Eva Kapsová, Jan Václavík

**Affiliations:** 1 Department of Internal Medicine and Cardiology, University Hospital Ostrava, 708 00 Ostrava, Czech Republic; marie.lazarova@fno.cz (M.L.); eva.kapsova@fno.cz (E.K.); jan.vaclavik@fno.cz (J.V.); 2 Department of Internal Medicine, Faculty of Medicine, University of Ostrava, 703 00 Ostrava, Czech Republic

**Keywords:** MELAS syndrome, heart failure (HF), mitochondrial disease, hypertrophic cardiomyopathy (HCM), genetic testing

## Abstract

**Background:** Mitochondrial encephalomyopathy with lactic acidosis and stroke-like episodes (MELAS) is a rare multisystem disorder caused by mitochondrial DNA mutations, most commonly the m.3243A>G variant in the MT-TL1 gene. Although neurological manifestations predominate, cardiac involvement, including hypertrophic cardiomyopathy (HCM), heart failure (HF), and arrhythmias, may be the initial or dominant presentation and often remains underrecognized. **Case Presentation:** We report a 43-year-old man with chronic kidney disease (CKD) and long-standing bilateral sensorineural hearing loss who presented with progressive dyspnea and acute decompensated HF. Transthoracic echocardiography revealed severe left ventricular (LV) systolic dysfunction with diffuse hypertrophy. Cardiac magnetic resonance showed non-ischemic cardiomyopathy with diffuse late gadolinium enhancement and increased LV wall thickness. Coronary angiography excluded obstructive disease. Initial endomyocardial biopsy performed at a referring center showed nonspecific hypertrophy and fibrosis without diagnostic features. Given the multisystem involvement, a metabolic or genetic etiology was suspected. Whole-exome sequencing identified the pathogenic m.3243A>G MT-TL1 mutation, confirming MELAS syndrome. The patient was managed with guideline-directed HF therapy, received an implantable cardioverter-defibrillator for primary prevention, and was subsequently evaluated for heart transplantation. **Conclusions:** This case highlights the importance of considering mitochondrial disorders in the differential diagnosis of unexplained cardiomyopathy, particularly when cardiac dysfunction coexists with renal impairment and auditory deficits. Comprehensive multimodality evaluation and genetic testing are essential to establishing a unifying diagnosis and optimizing management.

## 1. Introduction

Mitochondrial encephalomyopathy with lactic acidosis and stroke-like episodes (MELAS) syndrome is a rare multisystem disorder caused by pathogenic mutations in mitochondrial DNA (mtDNA), most frequently the m.3243A>G variant in the MT-TL1 gene [1,2]. MT-TL1 encodes mitochondrial transfer RNA for leucine, which is essential for oxidative phosphorylation and normal respiratory chain function.

From a cardiology perspective, mitochondrial cardiomyopathies represent a diagnostic challenge because their phenotypic spectrum overlaps with more prevalent conditions such as sarcomeric hypertrophic cardiomyopathy (HCM), infiltrative cardiomyopathies, and storage diseases. In many patients, cardiac involvement may dominate the clinical presentation for years before the development of classical neurological manifestations, leading to delayed or missed diagnosis. Importantly, early identification of a mitochondrial etiology has implications not only for patient management and prognosis but also for family screening due to maternal inheritance patterns.

Advances in multimodality imaging and genetic testing have substantially improved the recognition of rare metabolic cardiomyopathies. Cardiac magnetic resonance (CMR), in particular, allows for detailed tissue characterization and may reveal patterns of hypertrophy and fibrosis suggestive of mitochondrial disease. Nevertheless, awareness of these entities remains limited in routine clinical practice. This case therefore aims to highlight key diagnostic features and a practical diagnostic approach to unexplained HCM with multisystem involvement.

MELAS typically affects the central nervous system, skeletal muscle, kidneys, and auditory pathways; however, cardiac involvement is increasingly recognized [1,2,3]. Up to 20–40% of patients develop HCM, heart failure (HF), arrhythmias, or conduction abnormalities, often preceding neurological manifestations [3,4].

The diagnosis may be difficult due to the heterogeneous clinical presentation and overlap with more common cardiomyopathies. The concurrent presence of unexplained left ventricular (LV) hypertrophy, renal dysfunction, and sensorineural hearing loss should raise suspicion for an underlying mitochondrial disorder [1,4]. In such cases, genetic testing plays a key role in establishing a unifying diagnosis and guiding multidisciplinary management.

We present the case of a 43-year-old man with progressive HF, chronic kidney disease (CKD), and bilateral sensorineural hearing loss, in whom MELAS syndrome was diagnosed after an extensive diagnostic workup, highlighting the importance of considering rare multisystemic diseases in the differential diagnosis of unexplained HCM and HF.

## 2. Case Presentation

A 43-year-old man with a history of CKD (stage 3a) and long-standing bilateral sensorineural hearing loss requiring hearing aids since childhood presented with progressive dyspnea, orthopnea, and lower-limb edema. He had no history of diabetes, hypertension, or known cardiovascular disease. There were no prior neurological symptoms suggestive of stroke-like episodes, seizures, or encephalopathy.

**Figure 1:**
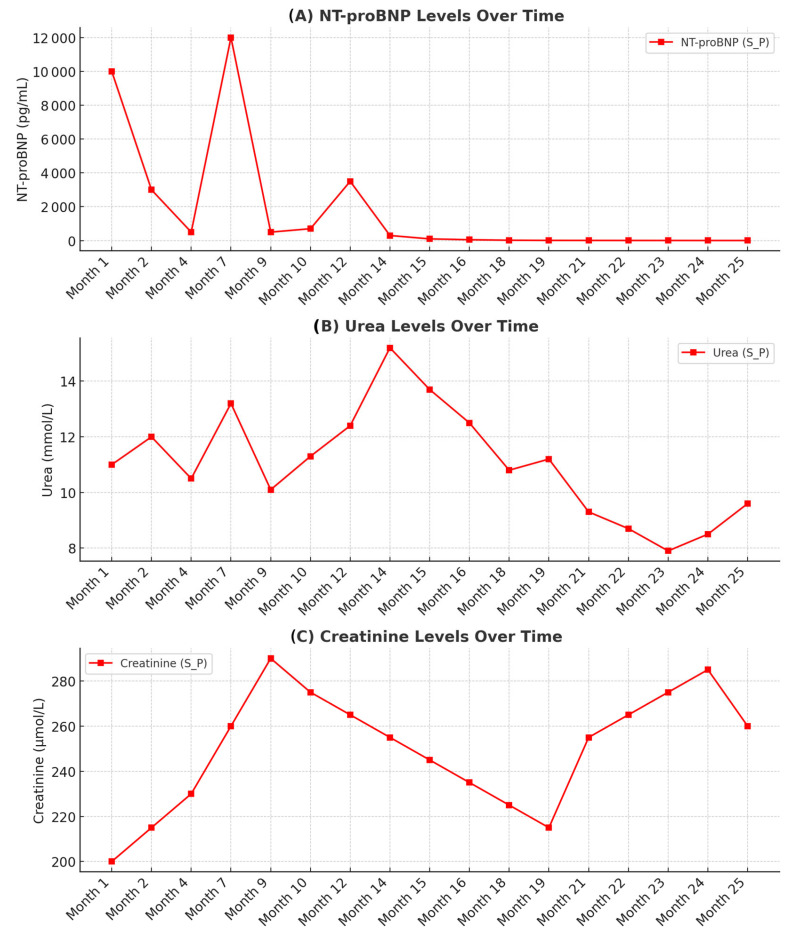
Dynamics of the laboratory tests during follow-up (**A**) NTproBNP, (**B**) serum urea, (**C**) serum creatinine. Values represent single measurements obtained at scheduled follow-up visits; no averaging (mean/median) was applied.

On admission, he was hypotensive (90/60 mmHg), tachycardic (120 bpm), and hypoxemic (89% on room air). Physical examination revealed bilateral basal crackles and signs of volume overload. Initial laboratory testing demonstrated markedly elevated NT-proBNP (5430 pg/mL), mild metabolic acidosis, and worsening renal function (creatinine 248 μmol/L; urea 12 mmol/L). Serum lactate measured at admission was mildly elevated (3.1 mmol/L). The longitudinal dynamics of key laboratory parameters during follow-up are summarized in Figure 1.

**Figure 2:**
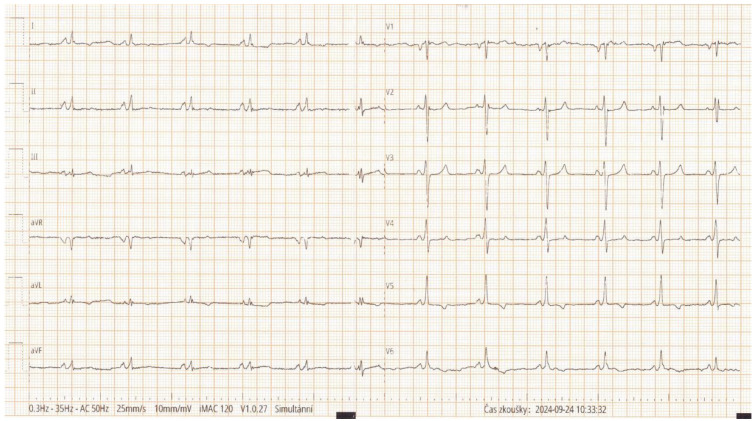
Electrocardiogram without voltage signs of left ventricular hypertrophy.

**Figure 3:**
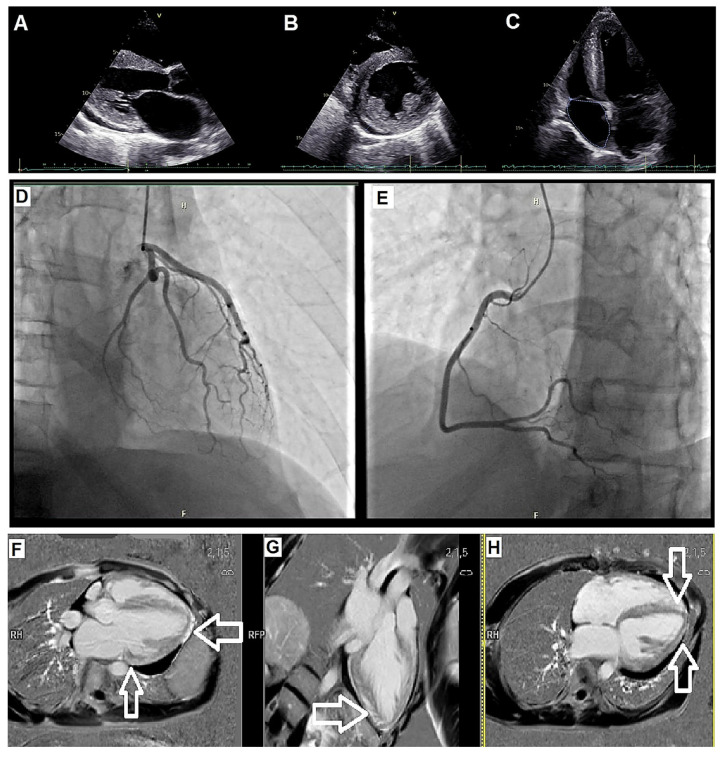
Echocardiography: (**A**) Left ventricular (LV) hypertrophy from Parasternal Long Axis (PLAX) with pericardial effusion behind the lateral wall. (**B**) LV hypertrophy from Parasternal Short Axis (PSA) with pericardial effusion. (**C**) LV hypertrophy from Apical 4 chamber (A4C). Negative selective coronary angiography: (**D**) Basin of the right coronary artery. (**E**) Basin of the left coronary artery. Cardiac Magnetic Resonance: (**F**) Arrows indicate diffuse left ventricular hypertrophy (LVH), mainly anteroseptal and lateral. Late gadolinium enhancement (LGE) shows replacement fibrosis, suggesting myocardial remodeling. (**G**) Arrow highlights hypertrophied myocardium in the basal anteroseptal and inferolateral segments. LGE confirms myocardial fibrosis with transmural involvement. (**H**) Arrows mark fibrotic areas in the basal and midventricular regions of the left ventricle. Transmural LGE suggests advanced myocardial damage and remodeling.

Electrocardiography showed sinus tachycardia without overt voltage criteria for left ventricular hypertrophy (Figure 2). Transthoracic echocardiography (TTE) revealed severe global LV systolic dysfunction with an ejection fraction (EF) of approximately 20%, diffuse hypokinesis, and concentric LV hypertrophy with a maximal wall thickness of 15 mm measured at the interventricular septum (Figure 3A–C). A small circumferential pericardial effusion was also noted.

Cardiac magnetic resonance (CMR) imaging demonstrated increased LV wall thickness (up to 15 mm), elevated LV mass index, and diffuse late gadolinium enhancement (LGE) involving the basal and mid-ventricular anteroseptal and inferolateral segments, consistent with non-ischemic cardiomyopathy with replacement fibrosis. Quantitative analysis confirmed the absence of regional ischemia and demonstrated a non-ischemic pattern of LGE distribution (Figure 3F–H).

Selective coronary angiography excluded obstructive coronary artery disease (Figure 3D,E). An endomyocardial biopsy (EMB) was performed at a referring center early during the diagnostic evaluation. Histology revealed nonspecific myocyte hypertrophy and interstitial fibrosis without diagnostic features; detailed micrographic documentation was not available for external review. Given the nondiagnostic biopsy findings and the coexistence of cardiac, renal, and auditory involvement, a multisystem disorder was suspected.

Given the combination of unexplained left ventricular hypertrophy, severe systolic dysfunction, CKD, and long-standing sensorineural hearing loss, an extensive differential diagnostic work-up was performed. Particular attention was paid to conditions associated with hypertrophic or infiltrative cardiomyopathy and systemic involvement.

Fabry disease was excluded by normal α-galactosidase A activity and the absence of characteristic findings on cardiac imaging. Transthyretin amyloidosis was considered unlikely based on negative bone scintigraphy and the absence of typical amyloid patterns on CMR imaging. Other lysosomal storage disorders, including Pompe disease and Alström syndrome, were also considered unlikely based on clinical phenotype and targeted testing.

Despite EMB demonstrating only nonspecific myocyte hypertrophy and interstitial fibrosis, the overall clinical picture raised strong suspicion of a metabolic or genetic disorder. Whole-exome sequencing was performed on peripheral blood DNA and identified the pathogenic m.3243A>G mutation in the MT-TL1 gene and confirming the diagnosis of MELAS syndrome. Genetic testing of myocardial tissue was not performed.

The patient was stabilized with intravenous diuretics and vasodilators, and guideline-directed medical therapy for HF was initiated, including beta-blockers (BB), mineralocorticoid receptor antagonists (MRA), and sodium-glucose co-transporter 2 (SGLT2) inhibitors. Sacubitril/valsartan was introduced after improvement in blood pressure. Coenzyme Q10 and L-arginine supplementation were added as supportive therapy for mitochondrial dysfunction based on current practice recommendations.

NT-proBNP was markedly elevated at baseline (5.430 pg/mL) and increased to 10.000 pg/mL within the first month. A further rise with a peak between months 4 and 9 coincided with a clinically documented HF decompensation requiring treatment intensification (Figure 1). Following stabilization, NT-proBNP gradually decreased in parallel with clinical improvement and optimization of HF therapy.

**Figure 4:**
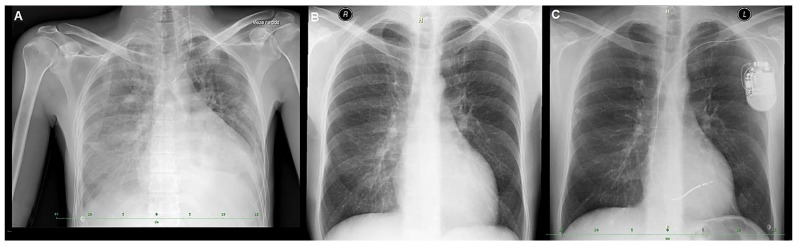
Anteroposterior chest X-ray. (**A**) Initial image with bilateral pleural effusion and signs of pulmonary edema. (**B**) Control image after pleural puncture on the left at month 1. (**C**) Control image after implantable cardioverter-defibrillator implantation at month 3.

Due to persistent LV dysfunction, the patient received a single-chamber implantable cardioverter-defibrillator (ICD) for primary prevention of sudden cardiac death (SCD) (Figure 4). A single-chamber ICD was selected because the patient had a narrow QRS (Figure 1) and no indication for atrial pacing, and to minimize additional hardware in the context of CKD and advanced HF management. He was subsequently evaluated by a multidisciplinary HF team and assessed for heart transplantation (HTx). Over 25 months of follow-up, he remained clinically stable without further hospitalizations. Genetic counseling was provided; however, further cascade testing was not feasible due to the absence of siblings and unavailable paternal information.

A timeline summarizing the key diagnostic and therapeutic milestones during the patient’s work-up and management is provided in Table 1.

## 3. Discussion

This case is noteworthy because MELAS syndrome presented with a predominantly cardiac phenotype and rapid progression to advanced HF, while overt neurological manifestations were absent at presentation. The diagnosis was established only after integrating extracardiac red flags with CMR tissue characterization and genetic testing, despite an initially nondiagnostic EMB.

MELAS syndrome is a rare mitochondrial disorder most commonly caused by the m.3243A>G mutation in the MT-TL1 gene, which encodes mitochondrial tRNA^Leu and is essential for mitochondrial protein synthesis and oxidative phosphorylation [1]. Although neurological involvement is classical, cardiac manifestations, including HCM, HF, arrhythmias, and conduction abnormalities, occur in up to 20–40% of patients and may precede neurologic symptoms [2,3,4,5]. In the present case, the patient exhibited no stroke-like episodes or seizures, and his dominant presentation was rapidly progressive HF, emphasizing the highly variable phenotype of MELAS.

The differential diagnosis of a hypertrophic phenotype is broad and includes numerous ‘phenocopies’ (e.g., infiltrative, storage, and mitochondrial diseases), for which a stepwise approach integrating clinical red flags, ECG, echocardiography, CMR tissue characterization, and genetics is recommended [6].

Diagnosing mitochondrial cardiomyopathy remains challenging due to its overlap with more common etiologies. The coexistence of unexplained LV hypertrophy, renal dysfunction, sensorineural hearing loss, and mildly elevated serum lactate in our patient prompted consideration of a multisystem disorder. In such settings, multimodality imaging plays a central role. CMR findings of diffuse LGE with a non-ischemic distribution pattern and increased LV mass strongly suggested a metabolic or mitochondrial etiology [7,8]. These features have been repeatedly described in mitochondrial cardiomyopathies and may provide diagnostic clues even in the absence of specific histological confirmation.

EMB may support the diagnosis of specific cardiomyopathy etiologies; however, in mitochondrial disease, the diagnostic yield can be limited and may depend on the availability of specialized analyses (e.g., mitochondrial-focused histochemistry and/or ultrastructural assessment). In the present case, biopsy performed at a tertiary center showed nonspecific findings and the detailed biopsy protocol (including any mitochondrial-specific staining or electron microscopy) was not available to the authors. Given the strong multisystem clinical suspicion, the nondiagnostic biopsy reinforced the need to proceed to genetic testing to establish a unifying diagnosis.

Whole-exome sequencing confirmed the m.3243A>G mutation in MT-TL1, providing definitive evidence of MELAS syndrome. Genetic testing was performed on peripheral blood DNA. In mtDNA disorders, heteroplasmy may vary substantially across tissues and may not directly reflect the degrees of organ involvement particularly between blood and post-mitotic tissues such as myocardium. Quantitative heteroplasmy data were not provided by the external laboratory in this case; therefore, we interpreted the confirmed pathogenic variant in the context of the patient’s multisystem phenotype and characteristic imaging findings. The presence of hearing loss since childhood and progressive renal impairment further supported a unifying mitochondrial etiology [9,10,11].

Management of MELAS-related cardiomyopathy is largely supportive. Our patient was treated with guideline-directed medical therapy for HF, including BB, MRA, SGLT2 inhibitors, and sacubitril/valsartan [12]. Coenzyme Q10 and L-arginine supplementation were added as adjunctive therapies, reflecting current practice in mitochondrial disorders, although robust clinical evidence remains limited [13,14,15,16]. Given persistent LV dysfunction, an ICD was implanted for primary prevention [17], in accordance with current recommendations. Ultimately, the patient was evaluated for HTx, which remains a consideration in advanced mitochondrial cardiomyopathy.

From a clinical standpoint, this case underscores several important lessons. First, mitochondrial disease should be considered in patients with unexplained cardiomyopathy when cardiac findings coexist with extracardiac features such as renal dysfunction or sensorineural hearing loss, even in the absence of neurological symptoms. Second, CMR plays a pivotal role in raising suspicion of a metabolic etiology through identification of diffuse, non-ischemic late gadolinium enhancement patterns.

Third, this case highlights the limitations of EMB in mitochondrial cardiomyopathies, particularly when specialized histochemical or ultrastructural analyses are not available. In such scenarios, genetic testing may provide the most definitive diagnostic information and should be considered early in the diagnostic pathway. Finally, establishing a unifying diagnosis enables appropriate genetic counseling, cascade testing, and long-term planning, including timely referral to advanced HF programs.

This case highlights the importance of considering mitochondrial disorders in patients with unexplained cardiomyopathy and multisystem involvement. Early genetic testing is particularly valuable when standard diagnostic evaluations are nondiagnostic. Family counseling is essential due to maternal inheritance, and cascade testing was recommended to the patient’s maternal relatives. The case underscores the diagnostic value of integrating clinical features, CMR findings, and genetic testing to establish a unifying diagnosis in complex multisystem presentations.

From an advanced HF perspective, mitochondrial disease raises additional considerations for HTx evaluation. Candidate assessment should explicitly address extracardiac involvement, particularly neurological status, renal function, and the overall trajectory of multisystem disease, because these factors may influence perioperative risk and post-transplant outcomes. In our patient, the severity of cardiac involvement prompted transplantation evaluation, while the extent and progression of extracardiac manifestations required careful multidisciplinary consideration.

Future research should focus on defining imaging and genetic markers that allow for the earlier identification of mitochondrial cardiomyopathies in cardiology practice. Prospective registries and collaborative studies are needed to better characterize the natural history, optimal heart failure management strategies, and transplant outcomes in this patient population. Increased awareness among cardiologists may ultimately lead to earlier diagnosis, improved patient selection for advanced therapies, and better integration of multidisciplinary care.

## 4. Conclusions

This case illustrates that MELAS syndrome may present predominantly with rapidly progressive cardiomyopathy and advanced HF, even in the absence of overt neurological manifestations. Integrating extracardiac ’red flags’ with CMR tissue characterization and early genetic testing can enable a unifying diagnosis and guide advanced HF management.

## Figures and Tables

**Table 1:** Timeline of key diagnostic and therapeutic procedures.

Timepoint	Key Findings	Diagnostic Procedures	Treatment/Decision
Baseline/prior history	CKD stage 3a; long-standing bilateral sensorineural hearing loss	-	-
Admission (Day 0)	Hypotension, tachycardia; HF decompensation; lactate mildly elevated	ECG, labs incl NT-proBNP, TTE	IV diuretics/vasoactive support (as applicable)
Early hospitalization	LV hypertrophy + systolic dysfunction; pericardial effusion	TTE (details), CMR	Guideline-directed HF therapy as tolerated
Etiologic work-up	Exclusion of CAD; suspicion of systemic disorder	Coronary angiography; Fabry testing (α-galactosidase A); EMB	-
Additional diagnostics	Nondiagnostic EMB (performed at a tertiary center)	EMB	-
Genetic confirmation	Pathogenic mtDNA variant consistent with MELAS	Whole-exome sequencing (peripheral blood)	MELAS diagnosis established
SCD prevention	Advanced HF with narrow QRS	Device selection	Single-chamber ICD implanted
Advanced HF pathway	Persistent severe LV dysfunction	Multidisciplinary evaluation	Evaluated for HTx
Family implications	Maternal inheritance possible	Counseling	Further family testing not feasible (no sibling; father unknown)

## Data Availability

The data presented in this study are available on request from the corresponding author. The data are not publicly available due to patient confidentiality, individual patient data are not publicly available.

## Abbreviations

The following abbreviations are used in this manuscript:

A4C	Apical 4 chamber
BB	beta-blockers
CKD	chronic kidney disease
CMR	cardiac magnetic resonance
EF	ejection fraction
EMB	endomyocardial biopsy
HCM	hypertrophic cardiomyopathy
HF	heart failure
HTx	heart transplantation
ICD	implantable cardioverter-defibrillator
LGE	late gadolinium enhancement
LV	left ventricular
LVH	left ventricular hypertrophy
MELAS	Mitochondrial encephalomyopathy with lactic acidosis and stroke-like episodes
MRA	mineralocorticoid receptor antagonists
mtDNA	mitochondrial DNA
PLAX	Parasternal Long Axis
PSA	Parasternal Short Axis
SCD	sudden cardiac death
SGLTi	sodium-glucose co-transporter 2 inhibitors
TTE	Transthoracic echocardiography
